# Metabolomics-Based Identification of Characteristic Phytogenic Components of Honey of Medicinal Plant *Amorpha fruticosa* L.

**DOI:** 10.3390/foods15081377

**Published:** 2026-04-15

**Authors:** Bin Zhang, Xinyu Wang, Dianli Yang, Yuqian Wu, Fanhua Wu, Jibo Zhang, Yan Wang, Ying Zhang, Ni Cheng, Haoan Zhao, Wei Cao

**Affiliations:** 1 College of Food Science and Technology, Northwest University, Xi’an 710069, China; 18841660520@163.com (B.Z.); wangxinyu983@163.com (X.W.); yangdianli_zz@163.com (D.Y.); 13363909656@163.com (Y.W.); wufanhua_1@163.com (F.W.); zjb1936819496@163.com (J.Z.); wy@techshake.cn (Y.W.); 20165024@nwu.edu.cn (Y.Z.); chengni@nwu.edu.cn (N.C.); 2 Bee Product Research Center of Shaanxi Province, Xi’an 710065, China

**Keywords:** phytogenic markers, metabolomics, *Amorpha fruticosa* L. honey, botanical authenticity, ononin, network pharmacology

## Abstract

To analyze the phytogenic components of honey of *Amorpha fruticosa* L. (AFH) and establish a targeted quantitative method, the liquid chromatography-mass spectrometry (LC-MS) based metabolomic technology was used in this study. Firstly, high performance liquid chromatography—quadrupole time-of-flight mass spectrometry (HPLC-QTOF-MS) untargeted metabolomics technology was used to screen candidate markers by comparing AFH metabolites with plant chemicals of *Amorpha fruticosa* L. Afterward, high performance liquid chromatography—triple quadrupole tandem mass spectrometry (HPLC-QQQ-MS/MS) was used to verify and identify the candidate markers, confirming ononin as the characteristic phytogenic marker of AFH, and determining its content range in AFH as 76.84–93.27 μg/kg (absent in acacia, rape, jujube, and *Galla chinensis* honey). Then, network pharmacology and molecular docking techniques were adopted to explore the gastric protective mechanism of ononin, and the results showed that ononin strongly binds AKT1 (binding free energy −8.0677 kcal/mol). Using the established method, the LC-MS analytical method for ononin in honey established in this study may be used for the authenticity identification of the characteristic phytogenic markers of AFH.

## 1. Introduction

Honey is favored by consumers worldwide due to its wide range of applications and various health-promoting effects. Among different types of honey, monofloral honey generally has a higher market value than multifloral honey because of its unique flavor and potential health benefits associated with specific nectar plants. Monofloral honey refers to honey produced by bees primarily collecting nectar from a single plant species (usually accounting for more than 45% of the nectar source), which is naturally ripened without artificial additives or extensive processing [1]. Current research and industry focus on monofloral honey mainly concentrate on its characteristic flavor [2] and bioactive components [3]. Due to differences in nectar plants, the flavor and active constituents vary among different monofloral honeys [4]. Therefore, identifying characteristic phytogenic markers in rare and distinctive monofloral honeys not only helps clarify the relationship between their chemical composition and health-promoting effects but also provides a scientific basis for quality-based pricing of honey, thereby promoting the high-quality development of the beekeeping industry.

*Amorpha fruticosa* L. Honey (AFH) is one of the rare and distinctive monofloral honeys in northern China [5]. It typically exhibits a purplish-red color and is also known as “blood honey.” Its nectar plant, *Amorpha fruticosa* L. (AF) [6], is a deciduous shrub of the Fabaceae family, native to North America [7], this species was introduced into China in the 1920s and has since been widely cultivated in the Yellow River basin, Yangtze River basin, and northeastern China, where it is mainly used for soil and water conservation, afforestation, and urban greening [8]. Meanwhile, it also provides remarkable ecological benefits, including windbreak, sand fixation, and soil amelioration. The medicinal potential of AF has attracted extensive attention from researchers worldwide. Various tissues of AF, including leaves, flowers, fruits, and seeds, are rich in diverse bioactive constituents, such as rotenoids, flavonoids, and phenylpropanoids [9]. Modern pharmacological studies have validated that this plant possesses multiple bioactivities, including antidiabetic, antioxidant, anti-inflammatory, antitumor, antimicrobial, and hepatoprotective effects [10]. Owing to its abundant bioactive metabolites and versatile pharmacological properties, AF has emerged as a promising hotspot in natural drug development. However, despite extensive pharmacological studies on AF, the characteristic phytogenic components of its honey remain largely unexplored. Studies have shown that AFH is rich in phenolic compounds, which not only have significant antioxidant activity [11] and a hepatoprotective effect, but also exhibit an excellent protective effect against ethanol-induced gastric injury. We have also preliminarily revealed that this activity is related to the regulation of the NF-κB signaling axis. However, the specific chemical components supporting its gastric protective effect are not yet clear. This research gap has seriously restricted the in-depth development and industrial upgrading of this characteristic honey [12]. Therefore, screening and identifying the characteristic phytogenic markers of AFH and exploring the molecular mechanism of its gastric protective effect are the core research goals of this paper.

Metabolomics has become an important tool for the characterization of complex food matrices and the screening of authenticity markers [13]. Common analytical platforms include gas chromatography (GC), gas chromatography–mass spectrometry (GC-MS), liquid chromatography–mass spectrometry (LC-MS), and nuclear magnetic resonance (NMR), each with distinct advantages and limitations in metabolite profiling [14]. GC-based methods are mainly suitable for volatile and thermally stable compounds [15], whereas NMR provides robust structural elucidation but suffers from relatively low sensitivity. In contrast, LC-MS offers high sensitivity, wide metabolite coverage, and excellent suitability for the detection of non-volatile and thermally labile phenolic compounds and flavonoids commonly present in honey [16].

Given that honey is rich in non-volatile plant-derived secondary metabolites, LC-MS-based metabolomics is particularly advantageous for the comprehensive screening and characterization of characteristic phytogenic markers. Therefore, LC-MS was selected in this study to enable high-resolution detection and facilitate the identification of potential marker compounds in AFH.

In light of this, the present study aims to establish a method based on metabolomics technology for discriminating characteristic phytogenic markers in AFH. The specific research strategy is as follows: Based on HPLC-QTOF-MS untargeted metabolomics, AFH samples were analyzed for component screening. Through feature peak extraction, ion fragment comparison, and database analysis, its phenolic composition was clarified. Subsequent comparison with the chemical constituents of the AF plant revealed that ononin is present in both AFH and its nectar plant, suggesting its potential as a characteristic phytogenic marker. HPLC-QQQ-MS/MS targeted metabolomics was then employed to establish a novel method for the qualitative and quantitative analysis of ononin in AFH. Applying this method to 24 batches of AFH samples, the ononin content was determined to range from 76.84 to 93.27 μg/kg. Notably, ononin was not detected in 24 batches of other honey samples (including acacia honey, rapeseed honey, jujube honey, and Galla chinensis honey). On the basis of clarifying the characteristic components, the mechanism was further explored by combining network pharmacology and molecular docking technology. The results showed that ononin had a high binding correlation with human AKT1 protein, suggesting that it may play a gastric protective role by regulating the AKT signaling pathway in coordination with the NF-κB signaling axis. This finding provides a clear molecular-level theoretical basis for explaining the known protective effect against ethanol-induced gastric injury of AFH. The LC-MS analysis method of ononin in honey established in this study may be used to authenticate the phytogenic authenticity of AFH. At the same time, it fills the gap in the study of the pharmacodynamic components of AFH and provides a scientific basis for the quality evaluation and functional development of bee products and other agricultural and forestry foods.

## 2. Materials and Methods

### 2.1. Chemicals and Reagents

Methanol (MeOH) and formic acid of LC/MS grade were purchased from Merck Co., Inc. (Rahway, NJ, USA). The ultrapure water (18.25 MΩ·cm) was obtained from the ULUPURE water purification system (Chengdu, China). Ethanol (analytical grade) and anhydrous ethanol (analytical grade) were obtained from Tianjin Kemiou Chemical Reagent Co., Ltd. (Tianjin, China). Ethyl acetate, hydrochloric acid, and anhydrous sodium sulfate (analytical grade) were purchased from Tianjin Fuchen Chemical Reagent Factory (Tianjin, China). Standard of ononin (HPLC grade, purity ≥ 99%) was purchased from Shanghai Yuanye Biotechnology Co., Ltd. (Shanghai, China). The 2,2-diphenyl-1-picrylhydrazyl Free Radical (DPPH) radical (analytical grade) was obtained from Merck KGaA (Darmstadt, Germany). Amberlite XAD-2 resin was purchased from The Dow Chemical Company (formerly Rohm and Haas Company, Spring House, PA, USA).

### 2.2. Honey Sample Production and Collection

Twenty-four batches of *Amorpha fruticosa* L. honey (AFH) samples were collected from different apiaries in Yulin (n = 6), Weinan (n = 6) of Shaanxi Province, and Cangzhou (n = 6), Qinhuangdao (n = 6) of Hebei Province, China. Other honey samples (n = 3 per type) were derived from different botanical origins, including acacia honey, jujube honey, rape honey, and Galla chinensis honey. All honey samples in this study were directly obtained from beekeepers during the flowering phenology of their respective nectar plants to ensure their authenticity, and were stored at 4 °C after being transported to the laboratory until analysis. Table 1 presents detailed information on the honey samples.

### 2.3. Melissopalynological Analysis

The melissopalynological analysis was performed according to Koelzer, K’s method with minor modifications [17]. 5 g diluted AFH was centrifuged at 10,000 rpm for 15 min to collect sediment. After the supernatant was discarded, the precipitate was repeatedly washed with distilled water and centrifuged three times to completely remove the interfering sugars. The washed precipitate was mixed, the sample was added to the blood cell counting plate, the cover glass was covered and the overflow was removed. First of all, the proportion of nectar plant pollen in 20 fields of view was randomly counted by using the bright field microscope (400×–1000×). According to the standard of monofloral honey, the proportion should be ≥45%. For further identification, another sample can be prepared, and the pollen spores can be morphologically observed and imaged using a scanning electron microscope at an appropriate resolution.

### 2.4. Physicochemical and In Vitro Antioxidant Activity Analysis

The physicochemical parameters of AFH, including moisture content, pH, amylase, 5-hydroxymethylfurfural (5-HMF), glucose, fructose, sucrose, proline, and total protein contents, were determined using standard or validated analytical methods. Moisture content, pH, amylase, 5-HMF, and the contents of glucose, fructose, and sucrose were determined according to AOAC-based methods [18]. Specifically, moisture content was measured using an Abbe refractometer; amylase was measured by spectrophotometry; 5-HMF content was determined by HPLC-DAD; and the contents of glucose, fructose, and sucrose were determined by HPLC-RID.

Proline content was determined according to the harmonized method of the International Honey Commission [19]. Briefly, 1 mL of AFH solution (0.1 g/mL) was mixed sequentially with formic acid and 3% ninhydrin solution, followed by reaction in a boiling water bath and then at 70 °C. After the addition of isopropanol, the absorbance was measured at 510 nm, and quantification was performed using a proline standard curve. Total protein content was determined using the Coomassie Brilliant Blue method [20]. Briefly, 2.5 g of the AFH sample was dissolved in water and diluted to 25 mL. Then, 2 mL of the sample solution was reacted with Coomassie Brilliant Blue reagent, and the absorbance was measured at 595 nm. Quantification was performed using a bovine serum albumin standard curve. Each sample was analyzed in triplicate, and the results were expressed as mean values.

The in vitro antioxidant activity of AFH was evaluated by determining total phenolic content (TPC), DPPH radical scavenging activity, ferric reducing antioxidant power (FRAP), and Fe^2+^ chelating activity. TPC was determined using the Folin–Ciocalteu method [21]. Briefly, 1 mL of AFH solution (0.1 g/mL) was mixed sequentially with 1 mL of Folin–Ciocalteu reagent, 5 mL of 1 mol/L Na_2_CO_3_ solution, and 3 mL of distilled water. After thorough mixing, the reaction mixture was incubated in the dark for 1 h, and the absorbance was measured at 760 nm. The results were expressed as mg gallic acid equivalents per kg of honey (mg GAE/kg); DPPH radical scavenging activity was determined according to the method of Brand-Williams et al. [22] with slight modifications. Briefly, 1 mL of DPPH methanolic solution (0.1 mg/mL) was mixed with 1 mL of AFH solution at different concentrations (0.0125, 0.025, 0.05, 0.1, and 0.2 g/mL) prepared in 95% ethanol. After incubation at room temperature in the dark for 1 h, the absorbance was measured at 517 nm using 95% ethanol as the blank. The half maximal inhibitory concentration (IC_50_) was calculated from the regression equation of concentration versus absorbance; FRAP was determined according to the method of Benzie and Strain [23]. Briefly, 0.4 mL of AFH solution (0.1 g/mL) was mixed with 3.6 mL of TPTZ working solution and incubated at 37 °C for 10 min. The absorbance was then measured at 593 nm. A calibration curve was prepared using FeSO_4_·7H_2_O standard solutions, and the results were expressed as μmol FeSO_4_·7H_2_O/g; Fe^2+^ chelating activity was determined according to the ferrozine method described by Dinis et al. [24]. Briefly, 200 μL of AFH solution (0.2 g/mL) was mixed sequentially with 100 μL of FeSO_4_ solution (1 mmol/L) and 300 μL of ferrozine solution (1 mmol/L). After mixing, 2.4 mL of methanol was added, and the mixture was allowed to react for 10 min. The absorbance was measured at 562 nm. A calibration curve was prepared using Na_2_EDTA standard solutions, and the results were expressed as mg Na_2_EDTA/g.

### 2.5. HPLC-QTOF-MS Analysis

The enrichment of phenolic compounds was conducted by solid-phase extraction (SPE) for isolating them from AFH samples [25]. Briefly, 20 g of honey was dissolved in 100 mL of acidified distilled water (pH adjusted to 2.0 with hydrochloric acid). The solution was subjected to wet loading, followed by rinsing with hydrochloric acid aqueous solution and distilled water to remove impurities. Subsequently, the target analytes were eluted with methanol, and the eluate was finally filtered through an organic phase filter membrane for HPLC-QTOF-MS analysis.

Separation was performed using an Agilent Poroshell 120 EC-C18 column (Agilent, Santa Clara, CA, USA) with an injection volume of 2 μL. The mobile phase consisted of ultrapure water (A) and methanol (B) at a flow rate of 0.3 mL/min, with the following linear gradient elution program: 0–3 min, 20% B; 3–12 min, 20–25% B; 12–16 min, 25–30% B; 16–25 min, 30–85% B; 25–28 min, 85–90% B. Mass spectrometry was conducted using an electrospray ionization (ESI) source in negative polarity mode. The drying gas temperature and flow rate were set at 300 °C and 10 L/min, respectively. The nebulizer pressure was 35 psi, while the sheath gas temperature and flow rate were maintained at 350 °C and 11 L/min, respectively [26].

### 2.6. HPLC-QQQ-MS/MS Analysis

First, 25.00 g of the honey sample was dissolved in 200 mL ultrapure water and transferred to a 500 mL funnel. The honey sample was extracted with ethyl acetate (100 mL each time) 3 times, 20 min each time. The organic phase was combined and filtered after adding 5.0 g anhydrous sodium sulfate to remove water. The filtrate was evaporated and concentrated to dryness at 45 °C. The extract was ultrasonically dissolved with 2 mL chromatographic methanol, and 1.5 mL solution was filtered through a 0.22 μm organic filter membrane for HPLC-QQQ-MS/MS analysis [27].

Accurately weighed 1.000 mg standard ononin was dissolved in methanol and diluted to 10 mL, and a 0.1 mg/mL stock solution was obtained. Then, methanol was used to gradually dilute the series of standard working solutions of 0.01, 0.05, 0.1, 0.2, and 0.5 μg/mL. The standard working solution concentration (*X*, μg/mL) was used as the abscissa, and the quantitative ion peak area (*Y*) was used as the ordinate. The standard curve was drawn and the regression equation and correlation coefficient (R^2^) were calculated.

The quantitative analyses of the targeted compound in honey samples were performed on an Agilent 1290 series HPLC system equipped with an Agilent Infinity II 6470 Triple Quad mass spectrometer (Agilent Technologies, Inc., Santa Clara, CA, USA). The chromatographic separation was performed on a Zorbax Eclipse Plus C18 column (50 × 2.1 mm, 1.8 μm) with an injection volume of 2 μL. The mobile phase was 0.1% formic acid aqueous solution (A) and chromatographic grade methanol (B) at a flow rate of 0.3 mL/min. The linear gradient elution program was: 0–4 min, 70–60% B; 4–5 min, 60–50% B; 5–7 min, 50–40% B; 7–10 min, 40–30% B; 10–13 min, 30–15% B; 13–16 min, 15% B; 16–18 min, 15–30% B; 18–20 min, 30–70% B; 20–21 min, 70% B. Mass spectrometry was performed using an electrospray ion source (ESI) in positive and negative polarity modes. The drying gas temperature and flow rate were set to 300 °C and 5 L/min, respectively. The nebulizer pressure was maintained at 45 psi, and the sheath gas temperature and flow rate were 250 °C and 11 L/min, respectively.

### 2.7. Method Validation

The HPLC-QQQ-MS/MS method established in this study was validated in terms of linearity, sensitivity, precision, and accuracy, following commonly used analytical validation approaches. Linearity was assessed using five concentration levels (0.01–0.5 μg/mL), and calibration curves were constructed by plotting peak area against concentration. The limit of detection (LOD) and limit of quantification (LOQ) were determined based on signal-to-noise ratios (S/N) of 3 and 10, respectively. Precision was evaluated in terms of intra-day and inter-day variability. Intra-day precision (repeatability) was assessed by analyzing spiked samples at two concentration levels (0.1 and 0.2 μg/mL) ten times within a single day, while inter-day precision (reproducibility) was determined over six consecutive days. Accuracy was evaluated by recovery experiments. Known amounts of ononin standard at three levels (0.1, 0.2, and 0.5 μg/mL) were spiked into AFH samples, and six parallel analyses were performed. Accuracy was evaluated based on recovery results using high-purity reference standards.

### 2.8. Statistical Analysis

All experiments were performed in triplicate and the results were shown as mean ± standard deviation (SD). In this study, SPSS 27.0 software (USA) was used to analyze the data results, and ANOVA analysis was used to evaluate the significance of the data. The peak extraction, qualitative identification and quantitative analysis of mass spectrometry data were performed by Agilent Mass Hunter Profinder B.10.0 software. Qualitative analysis was carried out by retention time and ion fragment information, and quantitative analysis was carried out by peak area. The content of ononin in AFH was calculated and quantified by Mass Hunter quantitative analysis B.10.0 software. Origin 2021 software is used to draw charts; the structure of the compound was drawn by ChemDraw 20.0 software.

### 2.9. Network Pharmacology and Molecular Docking Analysis

In order to explore whether ononin is involved in the regulation of gastric protection-related signaling pathways, this study used network pharmacology and molecular docking to verify [28]. Based on the active components of AFH identified by untargeted metabolomics (Table 2), this study focused on the characteristic component ononin. The canonical SMILES string of ononin was retrieved from the PubChem database, and its 3D structure was obtained for subsequent analysis. The crystal structure of human AKT1 protein was downloaded from the RCSB PDB database [29]. The protein pdb file was imported into PyMOL 2.5 software for pretreatment, including the removal of water molecules and extraneous small molecules. The 3D structure of ononin was constructed using Chem3D 20.0 software, underwent energy minimization treatment, and was saved in pdb format. The processed protein and ligand were imported into AutoDockTools 1.5.6 software for hydrogenation treatment and saved in PDBQT format. Finally, using PyMOL and MOE 2019 software, molecular docking and visualization analysis were carried out by the full-atom docking method. In order to systematically compare the binding affinity of ononin with different potential core target proteins, the minimum binding free energy (ΔG, kcal/mol) in each docking result was sorted out. Using data processing software (GraphPad Prism 9), the molecular docking binding energy heatmap was drawn with the target protein as the row and the binding energy value as the column.

## 3. Results

### 3.1. Melissopalynological Analysis

**Figure 1:**
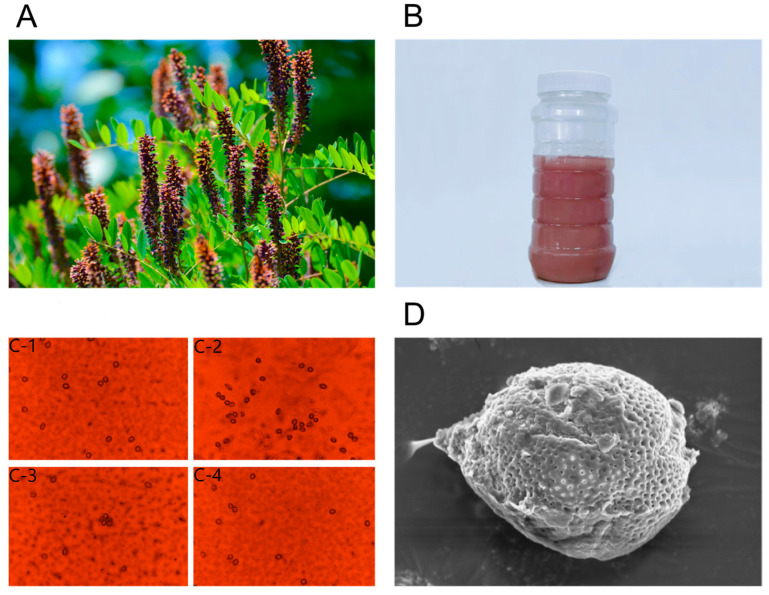
Melissopalynological analysis of *Amorpha fruticosa* L. honey (AFH). (**A**) The plant of *Amorpha fruticosa* L. (AF) and (**B**) the crystallized AFH. Typical light microscopy micrographs of dominant AF pollen grains in AFH from multiple fields of view (**C-1**–**C-4**), and (**D**) scanning electron microscopy micrograph of dominant AF pollen grains in AFH.

**Figure 2:**
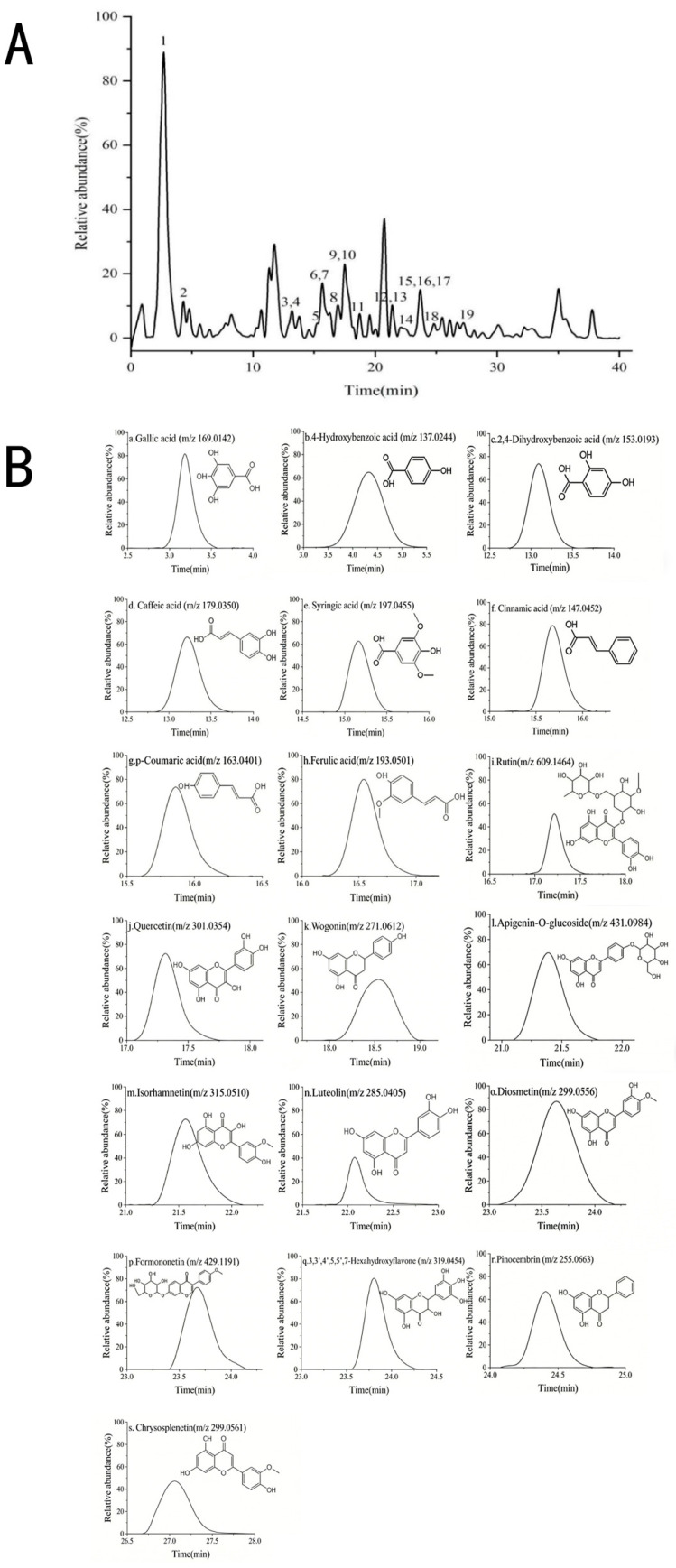
Metabolomics analysis of AFH by HPLC-QTOF-MS under negative ionization mode. (**A**) AFH phenolic compounds mass spectrometry negative ion mode total ion current diagram (TIC), The numbers correspond to the compounds listed in Table 3; (**B**) Extraction ion chromatogram (EIC) and structural formula of phenolic compounds identified in AFH in negative ion mode.

**Figure 3:**
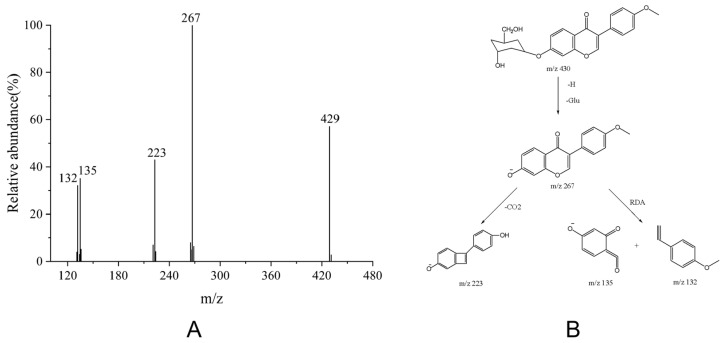
Secondary ion fragments and cleavage pathway of ononin in negative ion mode. (**A**) The secondary ion fragments of ononin in negative ion mode, the numbers indicate the precursor ion and major fragment ions of ononin in negative ion mode; (**B**) Fragmentation pathway of ononin in negative ion mode.

**Figure 4:**
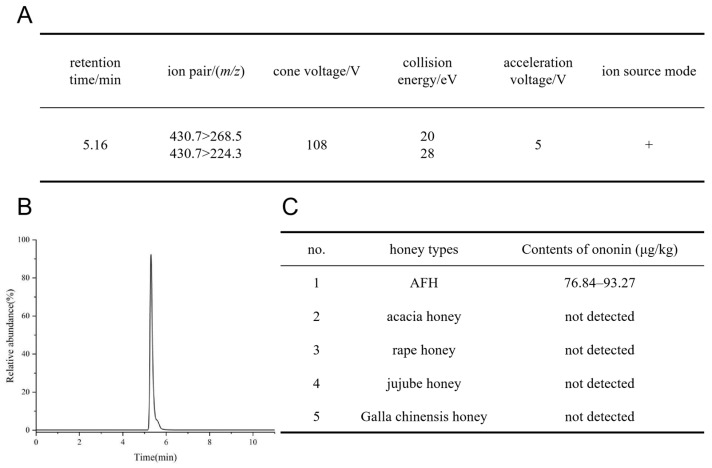
Metabolomics analysis based on HPLC-QQQ-MS/MS. (**A**) Retention time and mass spectrometry conditions of ononin; (**B**) MRM diagram of ononin standard solution; (**C**) the content of ononin in five kinds of honey.

Pollen in honey can directly act as a phytogenic marker for its botanical origin, but a small amount of exogenous pollen is inevitably mixed into honey during nectar collection and honey ripening. Thus, melissopalynological analysis is particularly critical for the traceability of nectar plants corresponding to Monofloral honey [30]. With melissopalynological characteristics analysis of AFH, as shown in Figure 1, crystallized AFH presents a purplish-red hue (Figure 1B). It is a general consensus that the content of dominant pollen in monofloral honey should exceed 45%, while honey with a dominant pollen content below 45% is defined as multifloral honey [31]. Under the optical microscope, AF pollen grains in the tested AFH were approximately circular with a basically uniform morphology (Figure 1(C-1–C-4)), and the relative content of AF pollen reached over 62%. This value meets the pollen ratio threshold for monofloral honey, and the morphological features were consistent with those of AF pollen grains recorded in the ICBB standard atlas. These results confirm that the collected AFH samples conform to the quality requirements of monofloral honey. Under a scanning electron microscope (SEM), the polar axis diameter of AF pollen grains was equivalent to the equatorial axis diameter (Figure 1D), as measured and calculated by image analysis software. The pollen grains exhibited the typical structural characteristics of AF pollen, which further provides a morphological basis for the melissopalynological identification of its nectar plant.

### 3.2. Physicochemical and In Vitro Antioxidant Activity Analysis

The physicochemical and antioxidant indices of the 24 AFH samples are summarized in Table 2. For comparison, the samples were classified into four groups according to production region: group A (Yulin, Shaanxi Province), group B (Weinan, Shaanxi Province), group C (Cangzhou, Hebei Province), and group D (Qinhuangdao, Hebei Province).

Physicochemical indices are widely used for honey quality evaluation [32]. Pita-Calvo et al. [33] noted in their review that moisture content, pH, amylase, 5-HMF, glucose, fructose, sucrose, proline, and total protein contents provide useful information on honey maturity, freshness, and overall quality. In the present study, moisture content ranged from 15.87 to 19.21%, pH from 3.68 to 4.07, amylase from 50.99 to 61.43 mL/g·h, 5-HMF from 0.75 to 2.08 mg/kg, glucose from 27.87 to 32.96 g/100 g, fructose from 42.33 to 46.38 g/100 g, sucrose from 1.04 to 2.94 g/100 g, proline from 280.67 to 377.89 mg/kg, and total protein from 624.15 to 896.74 mg/kg. Overall, the core physicochemical parameters of all AFH samples were within the acceptable ranges for honey quality evaluation [34].

Among these parameters, sugars are not only the major constituents of honey but are also closely associated with honey maturity and physicochemical behavior. The high glucose and fructose contents, together with the relatively low sucrose content, indicated a sugar profile characteristic of mature honey [35]. In addition, fructose content was consistently higher than glucose content in all AFH samples, and the fructose/glucose ratio ranged from 1.33 to 1.59, suggesting that AFH is less prone to crystallization under ambient conditions [36]. By contrast, proline and total protein contents showed more pronounced variation among the four groups. Previous studies by Sharma et al. [37] indicated that proline content in honey may be affected by bee species, nectar plant species, geographical environment, and climatic conditions. Accordingly, the differences observed in proline and total protein contents in the present study may also be associated with nectar plant growth conditions, production region, climate, and bee-related factors.

The TPC of AFH samples ranged from 247.27 to 302.33 mg GAE/kg, DPPH radical scavenging activity, expressed as IC_50_, ranged from 89.16 to 107.33 mg/mL, FRAP ranged from 1.73 to 2.36 μmol FeSO_4_·7H_2_O/g, and Fe^2+^ chelating activity ranged from 70.77 to 102.37 mg Na_2_EDTA/kg. Jaśkiewicz et al. [38] reported that these antioxidant-related indices are useful for characterizing the bioactive properties of honey and are often closely associated with differences in phenolic composition and content. Beretta et al. [39] reported that variation in the total phenolic content of honey from different production regions may be related to environmental factors such as climate, rainfall, and soil conditions, which may also partly explain the regional differences in antioxidant capacity observed in AFH. Overall, AFH exhibited measurable in vitro antioxidant activity, and the intergroup variation was at least partly associated with differences in phenolic constituents. These findings provide supportive evidence for the subsequent screening and identification of characteristic phytogenic components in AFH.

### 3.3. HPLC-QTOF-MS Analysis

Nectar plants are the main sources of phenolic acids and flavonoids in honey. These components are often transferred to honey through nectar transferred to honey via bee-collected nectar, which may become their characteristic phytogenic markers. In this study, solid-phase extraction (SPE) was used to enrich phenolic compounds in honey [40].

This method can effectively separate polar interfering substances such as carbohydrates, and has higher purity than Traditional Extraction Methods. HPLC-QTOF-MS was used for analysis. This technology combines the auxiliary qualitative ability of a diode array detector (DAD) and the accurate mass determination advantage of high-resolution mass spectrometry (HRMS), which can realize the efficient and accurate analysis of complex phenolic components [41].

The total ion chromatogram (TIC) of the phenolic extract of AFH collected in the negative ion mode is shown in Figure 2A. Within 0–40 min, each chromatographic peak exhibits a clear shape, good resolution, and no obvious overlap. The results indicate that the separation system and mass spectrometric detection conditions of HPLC-QTOF-MS are optimally adjusted, which can effectively capture the signals of phenolic metabolites in honey and provide reliable technical support for the subsequent qualitative identification of components [41]. After the raw data were processed by mass spectrometry analysis software, 19 bioactive phenolic compounds were predicted and identified based on molecular feature extraction, ion fragmentation patterns, and accurate matching of theoretically calculated ions. These compounds include phenolic acids and their derivatives (gallic acid, 4-hydroxybenzoic acid, 2,4-dihydroxybenzoic acid, caffeic acid, cinnamic acid, syringic acid, p-coumaric acid, ferulic acid) and flavonoids (quercetin, rutin, luteolin, isorhamnetin, diosmetin, chrysoeriol, naringenin, pinocembrin, myricetin, ononin, apigenin 7-O-glucoside) [42]. Flavonoids are further subdivided into flavones, flavanones, and flavonoid glycosides. The detailed mass spectrometric information and extracted ion chromatograms (EIC) of the predicted compounds are shown in Table 3 and Figure 2B, respectively.

As an important plant-derived bioactive component in honey, phenolic compounds are not only related to the nutritional value of honey but also have significant application value in honey authenticity identification. Previous studies have shown that there are significant differences in the composition and content of phenolic components in honey from different nectar plants, which can be used as the basis for nectar source identification [43]. The phenolic acids, such as gallic acid, 4-hydroxybenzoic acid, and syringic acid, as well as flavonoids, such as diosmetin, naringenin, and rutin, identified in this study are relatively common in bulk honey, and generally have different contents and proportions in different honeys. Notably, ononin, an isoflavonoid compound, was detected in AFH through LC-MS analysis. ALEXANDRA B et al. previously isolated ononin from the roots of AF [44], confirming its origin from the nectar plant. The detection of ononin, together with 18 other phenolic compounds, demonstrates that LC-MS-based metabolomics enables comprehensive profiling of phenolic components in AFH, providing reliable data for subsequent screening of characteristic phytogenic markers. The extracted ion chromatogram (EIC) of this compound is shown in Figure 2B.

The molecular ion [M − H]^−^ *m*/*z* of ononin is 429.1195. In the negative ion mode, it will first remove a molecule of glucose to obtain the most important molecular fragment [M − H-162]^−^, and its *m*/*z* is 267. Because isoflavonoids are non-cross-conjugated systems, when the 4′ position of the B ring is replaced by a methoxy group (as the B ring of ononin), the electron cloud density of the 3-position carbon connected to the 1′ position is increased. The secondary mass spectrometry shows that it continues to take off a molecule of CO_2_, and at the same time, the reverse Diels-Alder cleavage (RDA) reaction [45] occurs to generate fragments with *m*/*z* of 135 and 132. The secondary ion fragments of ononin in Negative Ion Mode are shown in Figure 3A, and the cleavage pathway is shown in Figure 3B.

These results indicate that the LC-MS method effectively identifies diverse phenolic compounds in AFH, supporting the rational selection of ononin as a candidate phytogenic marker for further targeted quantitative analysis.

### 3.4. HPLC-QQQ-MS/MS Analysis

To achieve accurate quantitative analysis of ononin in honey, the cone voltage of HPLC-QQQ-MS/MS was optimized to maximize the signal intensity of precursor ions. Meanwhile, the ion pair collision energy was optimized to maximize the signal intensity of qualitative and quantitative ions. Finally, multiple reaction monitoring (MRM) was used to monitor two pairs of ion pairs. The precursor ion, product ion, cone voltage, and collision energy of ononin are shown in Figure 4A. Figure 4B presents the Extracted Ion Chromatogram (EIC) and related mass spectrometric information of ononin.

The quantitative method established in this study exhibited good linearity. The calibration curve was expressed as *Y* = 536.43*X* + 47.78 (R^2^ = 0.9996), with a concentration range of 0.8–40 μg/kg, where *Y* is the corresponding peak area and *X* is the concentration of the standard stock solution (μg/mL). This indicates that the concentration has a good linear relationship with the peak area within the corresponding range. In this method, the signal-to-noise ratio (S/N) = 3 was used as the limit of detection (LOD), and S/N = 10 as the limit of quantitation (LOQ). The LOD of ononin was calculated to be 0.08 ng/mL, and the LOQ was 0.36 ng/mL. The intra-day (repeatability) and inter-day (reproducibility) relative standard deviations (RSDs) were 3.57–7.62%, indicating good precision of the method. The spiked recovery rate of ononin ranged from 85.67% to 94.29%, with the relative standard deviation less than 6.73%, demonstrating that it can satisfy the detection requirements. Detailed results are presented in Table 4. Therefore, the qualitative and quantitative analysis of ononin in honey samples based on the established HPLC-QQQ-MS/MS method is reliable [46].

According to the established method, the ononin in AFH was quantitatively analyzed. After calculation, the content of ononin in the measured AFH samples ranged from 76.84 to 93.27 μg/kg. The method was applied to detect 24 batches of honey samples from different producing areas in the laboratory, including acacia honey, rape honey, jujube honey and Galla chinensis honey, and analyzed under the same HPLC-MS conditions. The results showed that this component was only found in AFH and was not detected in other honeys. Therefore, we can consider ononin as a characteristic phytogenic component of AFH (Figure 4C).

### 3.5. Network Pharmacology and Molecular Docking Analysis

**Figure 5:**
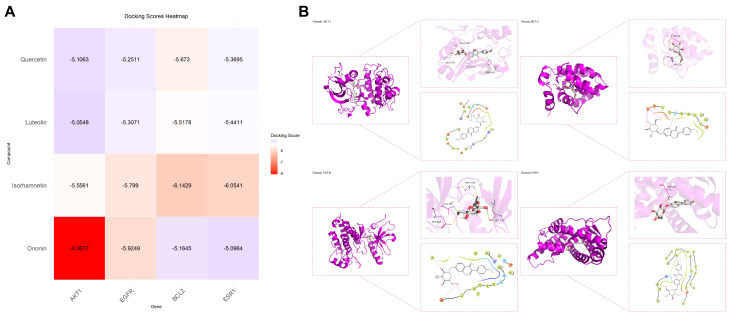
Molecular docking analysis of ononin with AKT1 protein. (**A**) Three-dimensional binding pose of ononin in the AKT1 active site, showing a binding energy of −8.07 kcal/mol; (**B**) Two-dimensional interaction map illustrating hydrogen bonds and hydrophobic contacts between ononin and AKT1 residues (Ile84, Arg273, Asp274).

In order to explain the potential role of ononin in the gastric protection of AFH, the binding ability of ononin to the key pathway node protein AKT1 was verified by molecular docking. As shown in Figure 5A, ononin can stably bind to the active pocket of AKT1 protein, and its binding free energy is −8.07 kcal/mol, indicating that there is a strong spontaneous binding tendency between the two. Further analysis of the binding mode showed that the aglycone and glycosyl part of ononin formed multiple hydrogen bond networks and hydrophobic interactions with key amino acid residues such as Ile84, Arg273, and Asp274 in the AKT1 active cavity (Figure 5B). The results confirmed at the structural level that ononin, a characteristic marker of AFH, could bind to AKT1 protein directly and with high affinity. The above results confirmed from the computational simulation level that the characteristic marker of AFH, ononin, can directly target and bind to the key pathway node AKT1 of gastric protection with high affinity. This provides a key molecular structure basis for explaining that ononin may play an anti-inflammatory and protective role in gastric mucosa by regulating AKT1 and its downstream NF-κB signaling axis [47].

## 4. Conclusions

In this study, based on LC-MS metabolomics analysis confirmed that ononin can be used as a plant-derived characteristic marker of AFH. The quantitative method constructed provides a reliable basis for its quality control and source identification, and fills the gap in the specific marker of the honey species. Further studies found that ononin can target AKT1, a key protein of gastric protection, with high affinity, which reveals the potential mechanism of anti-inflammatory and gastric mucosal protection of AFH by regulating AKT1-NF-κB signaling pathway at the molecular level, and provides important clues for its subsequent health efficacy research.

## Figures and Tables

**Table 1:** Sample information on honey samples from different botanical origins.

No.	Sample	Type of Honey	Botanical Source	Origin	Production Year
1	A1	Monofloral honey	*Amorpha fruticosa* L.	Yulin, Shaanxi Province	2021
2	A2	Monofloral honey	*Amorpha fruticosa* L.	Yulin, Shaanxi Province	2021
3	A3	Monofloral honey	*Amorpha fruticosa* L.	Yulin, Shaanxi Province	2021
4	A4	Monofloral honey	*Amorpha fruticosa* L.	Yulin, Shaanxi Province	2021
5	A5	Monofloral honey	*Amorpha fruticosa* L.	Yulin, Shaanxi Province	2021
6	A6	Monofloral honey	*Amorpha fruticosa* L.	Yulin, Shaanxi Province	2021
7	B1	Monofloral honey	*Amorpha fruticosa* L.	Weinan, Shaanxi Province	2021
8	B2	Monofloral honey	*Amorpha fruticosa* L.	Weinan, Shaanxi Province	2021
9	B3	Monofloral honey	*Amorpha fruticosa* L.	Weinan, Shaanxi Province	2021
10	B4	Monofloral honey	*Amorpha fruticosa* L.	Weinan, Shaanxi Province	2021
11	B5	Monofloral honey	*Amorpha fruticosa* L.	Weinan, Shaanxi Province	2021
12	B6	Monofloral honey	*Amorpha fruticosa* L.	Weinan, Shaanxi Province	2021
13	C1	Monofloral honey	*Amorpha fruticosa* L.	Cangzhou, Hebei Province	2022
14	C2	Monofloral honey	*Amorpha fruticosa* L.	Cangzhou, Hebei Province	2022
15	C3	Monofloral honey	*Amorpha fruticosa* L.	Cangzhou, Hebei Province	2022
16	C4	Monofloral honey	*Amorpha fruticosa* L.	Cangzhou, Hebei Province	2022
17	C5	Monofloral honey	*Amorpha fruticosa* L.	Cangzhou, Hebei Province	2022
18	C6	Monofloral honey	*Amorpha fruticosa* L.	Cangzhou, Hebei Province	2022
19	D1	Monofloral honey	*Amorpha fruticosa* L.	Qinhuangdao, Hebei Province	2021
20	D2	Monofloral honey	*Amorpha fruticosa* L.	Qinhuangdao, Hebei Province	2021
21	D3	Monofloral honey	*Amorpha fruticosa* L.	Qinhuangdao, Hebei Province	2021
22	D4	Monofloral honey	*Amorpha fruticosa* L.	Qinhuangdao, Hebei Province	2021
23	D5	Monofloral honey	*Amorpha fruticosa* L.	Qinhuangdao, Hebei Province	2021
24	D6	Monofloral honey	*Amorpha fruticosa* L.	Qinhuangdao, Hebei Province	2021
25	/	Monofloral honey	*Robinia pseudoacacia* L.	Weinan, Shaanxi Province	2023
26	/	Monofloral honey	*Robinia pseudoacacia* L.	Weinan, Shaanxi Province	2023
27	/	Monofloral honey	*Robinia pseudoacacia* L.	Weinan, Shaanxi Province	2023
28	/	Monofloral honey	*Robinia pseudoacacia* L.	Yan’an, Shaanxi Province	2020
29	/	Monofloral honey	*Robinia pseudoacacia* L.	Yan’an, Shaanxi Province	2020
30	/	Monofloral honey	*Robinia pseudoacacia* L.	Yan’an, Shaanxi Province	2020
31	/	Monofloral honey	*Brassica napus* L.	Deyang, Sichuan Province	2023
32	/	Monofloral honey	*Brassica napus* L.	Deyang, Sichuan Province	2023
33	/	Monofloral honey	*Brassica napus* L.	Deyang, Sichuan Province	2023
34	/	Monofloral honey	*Brassica napus* L.	Hanzhong, Shaanxi Province	2021
35	/	Monofloral honey	*Brassica napus* L.	Hanzhong, Shaanxi Province	2021
36	/	Monofloral honey	*Brassica napus* L.	Hanzhong, Shaanxi Province	2021
37	/	Monofloral honey	*Ziziphus jujuba* Mill.	Yulin, Shaanxi Province	2022
38	/	Monofloral honey	*Ziziphus jujuba* Mill.	Yulin, Shaanxi Province	2022
39	/	Monofloral honey	*Ziziphus jujuba* Mill.	Yulin, Shaanxi Province	2022
40	/	Monofloral honey	*Ziziphus jujuba* Mill.	Aksu, Xinjiang Uygur Autonomous Region	2021
41	/	Monofloral honey	*Ziziphus jujuba* Mill.	Aksu, Xinjiang Uygur Autonomous Region	2021
42	/	Monofloral honey	*Ziziphus jujuba* Mill.	Aksu, Xinjiang Uygur Autonomous Region	2021
43	/	Monofloral honey	*Rhus chinensis* Mill.	Hanzhong, Shaanxi Province	2022
44	/	Monofloral honey	*Rhus chinensis* Mill.	Hanzhong, Shaanxi Province	2022
45	/	Monofloral honey	*Rhus chinensis* Mill.	Hanzhong, Shaanxi Province	2022
46	/	Monofloral honey	*Rhus chinensis* Mill.	Yulin, Shaanxi Province	2023
47	/	Monofloral honey	*Rhus chinensis* Mill.	Yulin, Shaanxi Province	2023
48	/	Monofloral honey	*Rhus chinensis* Mill.	Yulin, Shaanxi Province	2023

Note: Samples A1–D6 represent *Amorpha fruticosa* L. honey. Other honey samples (Nos. 25–48) from different botanical origins were not coded and are indicated by “/”.

**Table 2:** Physicochemical properties and antioxidant activities of *Amorpha fruticosa* L. honey.

Sample	Moisture (%)	pH	Amylase (mL/g·h)	5-HMF (mg/kg)	Glucose (g/100 g)	Fructose (g/100 g)	Sucrose (g/100 g)	Proline Content (mg/kg)	Protein Content (mg/kg)	Total Phenolic Content mg GAE/kg	DPPH, IC_50_ mg/mL	FRAP,µmol FeSO_4_·7H_2_O/g	Fe(II), mg Na_2_EDTA/kg
A1	17.03 ± 0.06	3.91 ± 0.06	47.43 ± 0.05	ND	29.06 ± 0.05	44.47 ± 0.08	2.06 ± 0.03	289.75 ± 0.07	603.82 ± 0.08	298.76 ± 0.08	91.28 ± 0.04	2.17 ± 0.01	100.01 ± 0.12
A2	15.97 ± 0.08	3.89 ± 0.04	49.84 ± 0.05	ND	29.49 ± 0.06	44.41 ± 0.03	2.23 ± 0.08	296.85 ± 0.09	629.55 ± 0.08	286.52 ± 0.08	89.44 ± 0.02	2.26 ± 0.01	98.74 ± 0.05
A3	16.53 ± 0.11	3.87 ± 0.06	52.33 ± 0.04	ND	29.13 ± 0.09	43.71 ± 0.23	2.28 ± 0.06	289.33 ± 0.05	634.66 ± 0.06	293.44 ± 0.06	85.34 ± 0.04	2.34 ± 0.03	95.88 ± 0.04
A4	16.98 ± 0.13	3.77 ± 0.04	53.63 ± 0.03	ND	29.87 ± 0.08	45.20 ± 0.12	2.35 ± 0.07	280.76 ± 0.08	628.76 ± 0.08	280.53 ± 0.05	87.78 ± 0.04	2.16 ± 0.01	99.45 ± 0.05
A5	17.33 ± 0.10	3.81 ± 0.04	49.27 ± 0.05	0.75 ± 0.08 ^c^	28.76 ± 0.11	43.82 ± 0.07	2.34 ± 0.05	295.76 ± 0.07	629.58 ± 0.06	302.26 ± 0.08	88.67 ± 0.04	2.19 ± 0.02	94.54 ± 0.05
A6	16.67 ± 0.11	3.85 ± 0.05	53.46 ± 0.05	ND	27.97 ± 0.10	44.41 ± 0.06	2.06 ± 0.05	299.66 ± 0.08	618.55 ± 0.13	294.37 ± 0.04	92.46 ± 0.03	2.23 ± 0.01	102.34 ± 0.03
Group A mean	16.75 ± 0.48 ^c^	3.85 ± 0.05 ^a^	50.99 ± 2.52 ^c^	ND	29.05 ± 0.65 ^c^	44.34 ± 0.54 ^a^	2.22 ± 0.13 ^a^	292.02 ± 6.85 ^d^	624.15 ± 11.27 ^c^	292.65 ± 7.97 ^a^	89.16 ± 2.54 ^c^	2.22 ± 0.07 ^a^	98.49 ± 2.85 ^a^
B1	17.75 ± 0.10	4.02 ± 0.05	62.41 ± 0.03	ND	29.55 ± 0.10	43.76 ± 0.08	2.32 ± 0.04	326.75 ± 0.07	642.65 ± 0.07	298.45 ± 0.06	88.86 ± 0.04	2.32 ± 0.03	91.83 ± 0.04
B2	17.81 ± 0.15	3.96 ± 0.04	60.93 ± 0.06	ND	30.04 ± 0.07	43.55 ± 0.09	2.42 ± 0.03	335.83 ± 0.10	649.86 ± 0.07	283.39 ± 0.08	85.38 ± 0.05	2.02 ± 0.02	93.77 ± 0.04
B3	17.66 ± 0.07	3.84 ± 0.04	58.46 ± 0.03	ND	30.21 ± 0.08	42.77 ± 0.10	2.63 ± 0.05	338.47 ± 0.13	633.59 ± 0.12	283.93 ± 0.06	89.82 ± 0.07	2.12 ± 0.03	89.34 ± 0.05
B4	17.53 ± 0.23	3.91 ± 0.03	62.53 ± 0.05	1.86 ± 0.09 ^b^	30.24 ± 0.09	42.44 ± 0.11	2.89 ± 0.05	341.16 ± 0.12	627.37 ± 0.07	287.42 ± 0.09	91.83 ± 0.05	2.24 ± 0.03	86.92 ± 0.03
B5	17.64 ± 0.15	3.77 ± 0.04	61.77 ± 0.04	ND	31.43 ± 0.06	43.14 ± 0.10	2.17 ± 0.03	331.49 ± 0.08	631.55 ± 0.24	285.60 ± 0.10	87.48 ± 0.03	2.11 ± 0.03	89.92 ± 0.08
B6	17.1 ± 0.05	3.71 ± 0.02	62.48 ± 0.04	ND	30.48 ± 0.04	43.95 ± 0.07	2.71 ± 0.07	336.56 ± 0.08	646.14 ± 0.12	286.76 ± 0.07	93.88 ± 0.05	2.20 ± 0.01	88.74 ± 0.03
Group B mean	17.58 ± 0.26 ^b^	3.87 ± 0.12 ^a^	61.43 ± 1.58 ^a^	ND	30.33 ± 0.62 ^b^	43.27 ± 0.59 ^b^	2.52 ± 0.27 ^a^	335.04 ± 5.16 ^c^	638.53 ± 8.95 ^c^	287.59 ± 5.54 ^a^	89.54 ± 3.04 ^c^	2.17 ± 0.11 ^a^	90.09 ± 2.41 ^b^
C1	17.29 ± 0.06	3.83 ± 0.04	57.77 ± 0.05	ND	31.40 ± 0.15	42.92 ± 0.07	2.53 ± 0.07	364.35 ± 0.09	862.46 ± 0.11	272.38 ± 0.03	93.32 ± 0.07	1.88 ± 0.01	82.25 ± 0.05
C2	17.48 ± 0.06	3.72 ± 0.04	54.82 ± 0.07	ND	32.55 ± 0.11	44.12 ± 0.13	1.82 ± 0.06	372.54 ± 0.09	847.79 ± 0.13	269.33 ± 0.06	94.26 ± 0.06	1.75 ± 0.02	79.66 ± 0.05
C3	17.36 ± 0.11	3.76 ± 0.04	58.30 ± 0.04	ND	31.47 ± 0.06	44.65 ± 0.11	1.63 ± 0.06	367.67 ± 0.10	837.76 ± 0.20	263.48 ± 0.04	92.53 ± 0.05	1.79 ± 0.02	73.77 ± 0.04
C4	16.93 ± 0.06	3.83 ± 0.05	55.63 ± 0.05	ND	31.46 ± 0.08	43.55 ± 0.09	1.54 ± 0.06	364.83 ± 0.06	835.27 ± 0.10	259.91 ± 0.04	96.43 ± 0.06	1.83 ± 0.02	72.11 ± 0.08
C5	17.23 ± 0.11	3.94 ± 0.04	53.96 ± 0.07	2.08 ± 0.06 ^a^	31.83 ± 0.06	44.11 ± 0.11	1.83 ± 0.04	371.72 ± 0.05	842.33 ± 0.13	264.65 ± 0.08	97.26 ± 0.06	1.83 ± 0.02	70.84 ± 0.04
C6	17.03 ± 0.09	3.79 ± 0.04	57.21 ± 0.05	ND	32.29 ± 0.08	44.75 ± 0.08	1.43 ± 0.04	377.83 ± 0.06	872.45 ± 0.11	269.85 ± 0.08	92.17 ± 0.06	1.94 ± 0.01	78.77 ± 0.05
Group C mean	17.22 ± 0.21 ^b^	3.81 ± 0.08 ^a^	56.28 ± 1.74 ^b^	ND	31.84 ± 0.48 ^a^	44.02 ± 0.69 ^ab^	1.80 ± 0.39 ^b^	369.82 ± 5.19 ^a^	849.68 ± 14.76 ^b^	266.60 ± 4.68 ^b^	94.33 ± 2.09 ^b^	1.84 ± 0.07 ^b^	76.23 ± 4.62 ^c^
D1	18.35 ± 0.11	3.91 ± 0.02	59.68 ± 0.03	ND	30.60 ± 0.06	44.68 ± 0.09	1.43 ± 0.06	352.43 ± 0.07	862.69 ± 0.17	251.13 ± 0.11	103.41 ± 0.10	1.96 ± 0.04	73.82 ± 0.05
D2	18.61 ± 0.07	3.77 ± 0.04	58.33 ± 0.06	ND	29.76 ± 0.07	44.30 ± 0.06	1.75 ± 0.06	358.92 ± 0.04	902.32 ± 0.12	258.36 ± 0.07	105.46 ± 0.04	2.04 ± 0.04	70.81 ± 0.04
D3	18.88 ± 0.05	3.86 ± 0.07	60.43 ± 0.05	ND	31.42 ± 0.05	43.44 ± 0.10	1.80 ± 0.05	362.65 ± 0.05	873.62 ± 0.32	247.34 ± 0.06	103.56 ± 0.04	1.87 ± 0.03	71.87 ± 0.05
D4	18.73 ± 0.04	3.73 ± 0.03	58.43 ± 0.05	ND	32.85 ± 0.11	43.83 ± 0.06	1.38 ± 0.03	364.50 ± 0.07	937.56 ± 0.33	253.77 ± 0.05	113.43 ± 0.06	1.79 ± 0.03	73.93 ± 0.03
D5	18.70 ± 0.06	3.91 ± 0.03	57.82 ± 0.04	ND	31.66 ± 0.07	46.31 ± 0.08	1.30 ± 0.06	367.79 ± 0.05	921.64 ± 0.26	258.60 ± 0.14	108.89 ± 0.06	1.86 ± 0.01	72.05 ± 0.05
D6	19.13 ± 0.09	3.76 ± 0.02	56.32 ± 0.04	ND	30.47 ± 0.05	45.77 ± 0.09	1.13 ± 0.09	365.29 ± 0.06	882.59 ± 0.14	249.80 ± 0.07	109.20 ± 0.04	1.87 ± 0.03	74.34 ± 0.04
Group D mean	18.73 ± 0.80 ^a^	3.82 ± 0.08 ^a^	58.50 ± 1.44 ^b^	ND	31.13 ± 1.09 ^ab^	44.72 ± 1.12 ^a^	1.47 ± 0.26 ^b^	361.93 ± 5.51 ^b^	896.74 ± 29.02 ^a^	253.17 ± 4.61 ^c^	107.33 ± 3.90 ^a^	1.90 ± 0.09 ^b^	72.80 ± 1.42 ^c^

Note: For individual samples, values are expressed as mean ± SD of triplicate determinations (n = 3); for group means, values are expressed as mean ± SD of six honey samples per group (n = 6). Different lowercase letters within the same column indicate significant differences among the four groups (*p* < 0.05). Values without lowercase letters indicate no significant difference. ND, not detected. A1–A6, Yulin, Shaanxi Province; B1–B6, Weinan, Shaanxi Province; C1–C6, Cangzhou, Hebei Province; D1–D6, Qinhuangdao, Hebei Province.

**Table 3:** Mass spectrometry information of phenolic compounds of *Amorpha fruticosa* L. honey.

No.	Tentative Identification	Retention Time (min)	Molecular Formula	Calc (*m*/*z*)	[M − H]^−^ (*m*/*z*)	Error (ppm)	Fragment Ions (*m*/*z*)
1	Gallic acid	3.188	C_7_H_6_O_5_	169.0142	169.0153	−6.51	169, 125
2	4-Hydroxybenzoic acid	4.234	C_7_H_6_O_3_	137.0244	137.0241	2.19	119, 93
3	2,4-Dihydroxybenzoic acid	13.073	C_7_H_6_O_4_	153.0193	153.0191	1.31	153, 109
4	Caffeic acid	13.206	C_9_H_8_O_4_	179.0350	179.0334	8.94	161, 135
5	Syringic acid	15.150	C_9_H_10_O_5_	197.0455	197.0448	3.55	179, 153
6	Cinnamic acid	15.648	C_9_H_8_O_2_	147.0452	147.0447	3.40	129, 103
7	p-Coumaric acid	15.848	C_9_H_8_O_3_	163.0401	163.0394	4.29	119
8	Ferulic acid	16.529	C_10_H_10_O_4_	193.0501	193.0489	6.22	178, 149
9	Rutin	17.210	C_27_H_30_O_16_	609.1464	609.1457	1.15	301
10	Quercetin	17.310	C_15_H_10_O_7_	301.0354	301.0346	2.66	301, 283, 273, 151
11	Naringenin	18.523	C_15_H_12_O_5_	271.0612	271.0622	-3.69	253, 243, 151, 119
12	Apigenin 7-O-glucoside	21.364	C_21_H_20_O_10_	431.0984	431.0976	1.86	431, 269
13	Isorhamnetin	21.546	C_16_H_12_O_7_	315.0510	315.0492	5.71	315, 300, 271
14	Luteolin	22.078	C_15_H_10_O_6_	285.0405	285.0391	4.91	257, 151
15	Diosmetin	23.623	C_16_H_12_O_6_	299.0556	299.0541	5.02	281, 271, 255, 151
16	Ononin	23.673	C_22_H_22_O_9_	429.1191	429.1195	−0.93	267, 223, 135, 132
17	Myricetin	23.789	C_15_H_12_O_8_	319.0454	319.0447	2.19	319, 301, 167, 151
18	Pinocembrin	24.404	C_15_H_12_O_4_	255.0663	255.0650	5.10	237, 227, 221, 151
19	Chrysoeriol	27.029	C_16_H_12_O_6_	299.0561	299.0543	6.02	281, 271, 151

**Table 4:** Method parameters for the determination of *Amorpha fruticosa* L. honey by HPLC-QQQ-MS/MS.

Parameters	Results
concentration range (μg/mL)	0.01–0.5
linearity regression equation	*Y* = 536.43*X* + 47.78
correlation coefficient, R^2^	0.9996
sensitivity	
limit of detection (LOD) (ng/mL)	0.08
limit of quantitation (LOQ) (ng/mL)	0.36
spiked recovery rate (1.00 μg/mL)	0.8567–0.9429
stability	
intraday relative standard deviation (RSD) (1.00 μg/mL, 0.2 μg/mL)	3.57% (0.1 μg/mL), 5.26% (0.2 μg/mL)
interday relative standard deviation (RSD) (1.00 μg/mL, 0.2 μg/mL)	5.41% (0.1 μg/mL), 7.62% (0.2 μg/mL)

## Data Availability

The original contributions presented in the study are included in the article; further inquiries can be directed to the corresponding authors.
